# Comparative genomics provides insights into the lifestyle and reveals functional heterogeneity of dark septate endophytic fungi

**DOI:** 10.1038/s41598-018-24686-4

**Published:** 2018-04-20

**Authors:** Dániel G. Knapp, Julianna B. Németh, Kerrie Barry, Matthieu Hainaut, Bernard Henrissat, Jenifer Johnson, Alan Kuo, Joanne Hui Ping Lim, Anna Lipzen, Matt Nolan, Robin A. Ohm, László Tamás, Igor V. Grigoriev, Joseph W. Spatafora, László G. Nagy, Gábor M. Kovács

**Affiliations:** 1 Department of Plant Anatomy, Institute of Biology, Eötvös Loránd University, Budapest, 1117 Hungary; 2 US Department of Energy (DOE) Joint Genome Institute, Walnut Creek, CA 94598 United States; 3 Architecture et Fonction des Macromolécules Biologiques (AFMB), UMR 7257 CNRS Université Aix-Marseille, 13288 Marseille, France; 4 INRA, USC 1408 AFMB, 13288 Marseille, France; 5 Department of Biological Sciences, King Abdulaziz University, Jeddah, 21589 Saudi Arabia; 6 Microbiology, Department of Biology, Faculty of Science, Utrecht University, Utrecht, The Netherlands; 7 Department of Plant Physiology and Molecular Plant Biology, Institute of Biology, Eötvös Loránd University, Budapest, 1117 Hungary; 8 Department of Plant and Microbial Biology, University of California Berkeley, Berkeley, CA 94720 USA; 9 Department of Botany and Plant Pathology, Oregon State University, Corvallis, United States; 10 Synthetic and Systems Biology Unit, Institute of Biochemistry, BRC-HAS, Szeged, 6726 Hungary; 11 Plant Protection Institute, Centre for Agricultural Research, Hungarian Academy of Sciences, Budapest, 1022 Hungary

## Abstract

Dark septate endophytes (DSE) are a form-group of root endophytic fungi with elusive functions. Here, the genomes of two common DSE of semiarid areas, *Cadophora* sp. and *Periconia macrospinosa* were sequenced and analyzed with another 32 ascomycetes of different lifestyles. *Cadophora* sp. (Helotiales) and *P. macrospinosa* (Pleosporales) have genomes of 70.46 Mb and 54.99 Mb with 22,766 and 18,750 gene models, respectively. The majority of DSE-specific protein clusters lack functional annotation with no similarity to characterized proteins, implying that they have evolved unique genetic innovations. Both DSE possess an expanded number of carbohydrate active enzymes (CAZymes), including plant cell wall degrading enzymes (PCWDEs). Those were similar in three other DSE, and contributed a signal for the separation of root endophytes in principal component analyses of CAZymes, indicating shared genomic traits of DSE fungi. Number of secreted proteases and lipases, aquaporins, and genes linked to melanin synthesis were also relatively high in our fungi. In spite of certain similarities between our two DSE, we observed low levels of convergence in their gene family evolution. This suggests that, despite originating from the same habitat, these two fungi evolved along different evolutionary trajectories and display considerable functional differences within the endophytic lifestyle.

## Introduction

The vast majority of land plants are known to form symbioses with diverse fungal endophytes. These are fungi that, during some point of their life cycle, colonize plant tissues without causing symptoms of tissue damage^1–4^. Apart from behaving as commensalistic symbionts, fungal endophytes can also act as latent pathogens, latent saprotrophs, and mutualistic symbionts^2,5^. Although colonization by these fungi can be restricted to aboveground tissues, as in the case of clavicipitaceous endophytes^6^, roots can also be colonized by a broad spectrum of fungal endophytes with potentially diverse functions^7–10^.

The presence of root endophytes with melanized and septated intraradical hyphae has been known for over a century^11^. Despite these fungal endophytes dominating several biomes and climatic regions, their functions in relation to plants and the greater ecosystem are still elusive^12^. Even though this form-group is known as dark septate endophytes (DSE)^11,13^, varying degrees of melanization can be found in some of these fungi and some can actually form hyaline structures either^14^. DSEs, the ‘Class 4 endophytes’ *sensu* Rodriguez *et al*.^10^, are mostly asexual filamentous ascomycetes belonging to diverse lineages of numerous orders, including Helotiales, Xylariales and Pleosporales^8,11,15–18^. Although DSEs can be found worldwide, they are more frequent in harsh, nutrient-limited environments such as arid and semiarid areas^12,18,19^. Their prevalence suggests that their importance in these ecosystems might be crucial. Several aspects of the lifestyle, ecology, and evolution of DSEs, and their mode of interaction with plants is not well understood^8^. Studies in experimental systems have suggested that fungal root endophytes could be latent pathogens^10,20^. The effects of root-colonizing endophytes on their hosts depends on the ontogenetic, physiologic and genotypic status of the host, as well as on the availability of organic/inorganic nutrients and environmental/experimental conditions^2,10,17,21^. Various degradative enzyme activities have been detected in DSEs^22,23^, which could indicate that they have a rich plant cell wall degrading enzyme (PCWDE) repertoire. Thus, DSEs could be important as (latent) saprobes, and also play a role in host nutrition through complex substrate degradation. DSEs might help to degrade organic matter in nutrient-poor soils in a similar way as ericoid mycorrhizal fungi – mutualistic symbionts that benefit the host plant by mobilizing complex substrates in nutrient poor environments^24^.

Root-associated fungi and DSE communities of (semi−) arid environments and grasslands have been studied in detail and the results suggest that there are core members of those communities common to disparate regions^16,25–27^. Our knowledge of the biology and functions of DSEs in those environments is limited compared to those of other DSEs such as the helotialean root endophytes of woody plants (especially conifers) like the *Phialocephala fortinii* s.l. – *Acephala applanata* species complex (PAC)^28^, and the genome of one member of this complex was sequenced recently^29^. Although the biogeographic distribution of DSEs is not properly understood, it seems that the DSE communities of forest ecosystems remarkably differ from those of grasslands, e.g., we are not aware of any PAC fungi colonizing plants in grassland ecosystems.

Although comparative genomics could provide insights into fundamental biological and evolutional questions, to date, only few genomes of taxonomically distinct endophytic fungi became available. These include the genomes of clavicipitaceous shoot/systemic endophytes^6,30^, other non-root colonizing fungi like *Xylona heveae*^31^, *Pestalotiopsis fici*^32^ and *Phialocephala scopiformis*^33^, and root endophytes including *Serendipita indica*^34^ (formerly *Piriformospora indica*, Sebacinales, Basidiomycotina), *Colletotrichum trifolium*^35^ and the DSE fungi *Harpophora oryzae*^36^, *Phialocephala subalpina*^29^ and *Microdochium bolleyi*^37^. These endophytic fungi have highly distinct genomic toolboxes, diverse ancestral lifestyles and different habitats. Therefore, based on currently available genomic information, it is almost impossible to construct an overall view of fungal endophytic lifestyle. Further data is needed to uncover common genomic features, like in the case of the ectomycorrhizal (EcM) lifestyle that independently arose multiple times during evolution from saprotrophic ancestors^38^. The EcM lifestyle is associated with the loss of PCWDE-encoding genes and with the diversification of lineage-specific symbiosis-related genes^38–41^. In contrast to ectomycorrhizal fungi, the endophytic lifestyle of ascomyceteous root colonizers such as *C. tofieldiae*, *H. oryzae* and *P. subalpina*, is not accompanied by a reduction in the PCWDE repertoire^5,29,35,36^.

Here, we perform in-depth analyses of the genomes of two dominant DSEs that originate from semiarid grasslands. These fungi – *Cadophora* sp. and *Periconia macrospinosa* – albeit from the same environment, represent taxonomically distant species with different host preferences^16,18^. As these species are common and widespread members of DSE communities of semiarid sandy grasslands^16,19,42^, they likely play key roles in the functioning of such ecosystems. We analyze their repertoires of CAZymes, PCWDEs and other relevant gene families and compare these to that of other ascomycetes to understand whether independently evolved DSE lineages possess common genomic signatures and to gain insights into their lifestyle. To the best of our knowledge, this is the first comparative genome analysis of two different fungal root endophytes that belong to the same habitat.

## Materials and Methods

### Fungal strains used for genome sequencing

We analyzed two DSEs: *Cadophora* sp. (strain DSE1049) and *P. macropsinosa* (strain DSE2036). *Cadophora* sp. DSE1049 represents a helotialean root endophyte that mainly colonizes non-gramineous plants^16,43^. It has been detected all over Europe^43^ and in other diverse geographic regions including Antarctica^44,45^ and Argentina^46^. *Cadophora* sp. DSE1049 is not conspecific with any described species of the *Cadophora* genus which comprises several root endophytes including *C. finlandica* and *C. orchidicola*^18^. The strain DSE1049 was isolated from healthy roots of *Salix rosmarinifolia* from grasslands near Fülöpháza, Hungary^16^. *P. macrospinosa*, on the other hand, is a well-known pleosporalean root colonizer belonging to *Periconiaceae*, Pleosporales^47^. This taxa is one of the most dominant and widespread DSEs in grassland ecosystems worldwide, and is associated with gramineous plants^12,16,48^. The strain DSE2036 was isolated from healthy roots of *Festuca vaginata* from the same site as mentioned above^16,42^.

**Figure 1:**
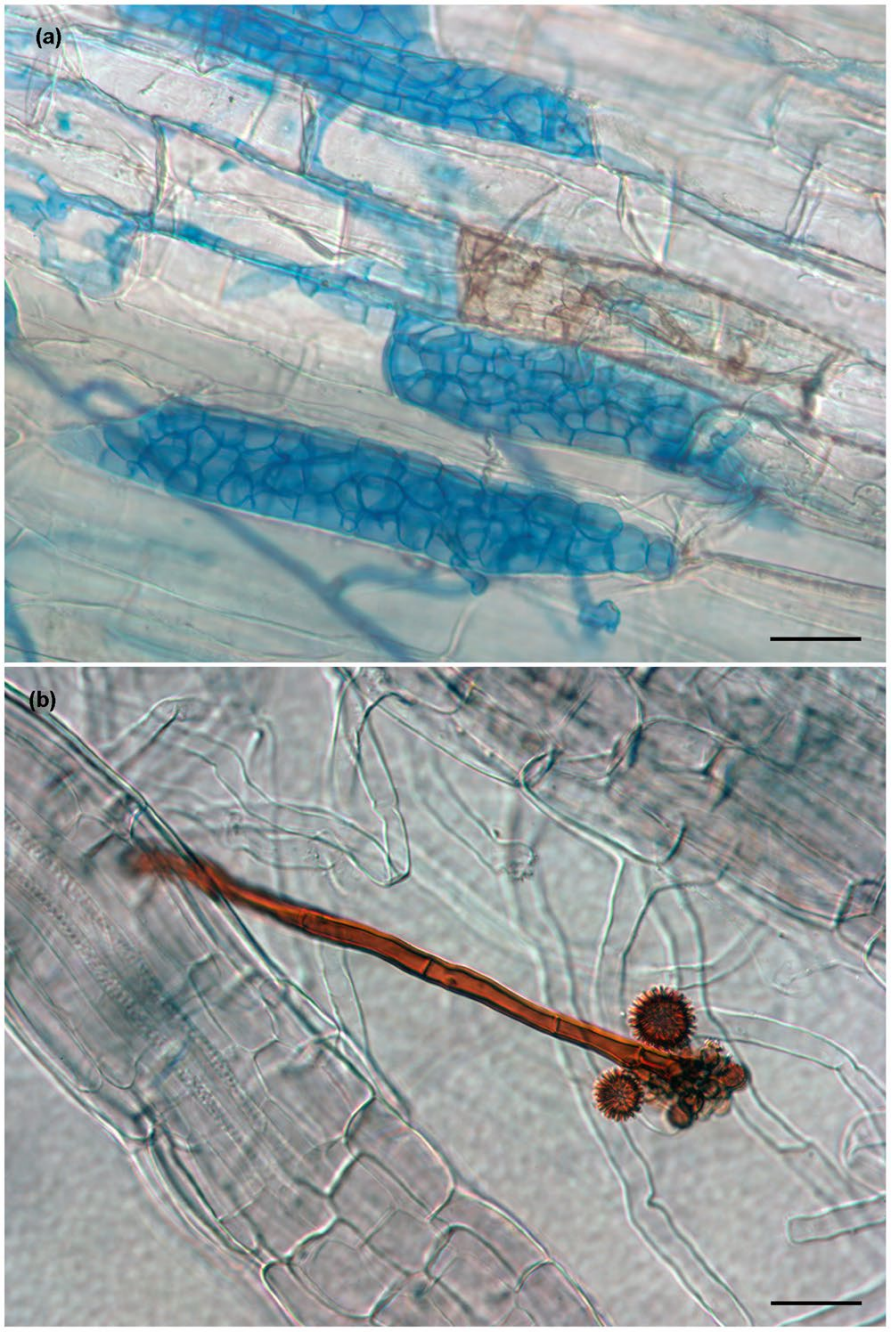
Characteristic structures of *Cadophora* sp. and *Periconia macrospinosa* in an artificial inoculation system with *Zea mays*. (**a**) Intra- and intercellular septate hyphae and microsclerotia of *Cadophora* sp. are visualized after staining with aniline blue. Intracellular pigmented hyphal structures in the root can also be seen. (**b**) *P. macrospinosa* colonization of roots with barely stainable hyphae. Its pigmented conidiophore can be seen with characteristic spiny conidia. Scale bars 30 µm.

Both species has melanized hyphae in culture and brownish isolates. Both species colonized leek and maize roots in *in vitro* experiments, formed intraradical structures (e.g. like microsclerotia (Fig. 1a)) characteristic of DSEs^16^. Intraradical hyphae of *Cadophora* sp. DSE1049 are typically dark, and can easily be stained by common fungal blue dyes (Fig. 1a). In contrast, *P. macrospinosa* usually have hyaline intraradical hyphae that cannot be visualized by typical fungi stains, and within the roots, only certain structures (e.g. microsclerotia, chlamydospores and conidiophores) are melanized (Fig. 1b)^16,49,50^.

### Nucleic acid extraction

*Cadophora* sp. DSE1049 and *P. macrospinosa* DSE2036 were maintained in modified Melin-Norkrans (MMN) liquid medium^51^ containing 3 g/L glucose and were grown as a free-living vegetative mycelium for two weeks at room temperature in the dark. For harvesting, the mycelium was dried on filter paper, flash-frozen in liquid nitrogen and ground into a powder. DNA was extracted from 2.5 g mycelium using the DNeasy Plant Maxi kit (Qiagen) according to the manufacturer’s instructions (doing on-column RNase treatment). In addition, total RNA was isolated from 0.5 g mycelium using the RNeasy Plant Midi Kit (Qiagen) according to the manufacturer’s instructions, including DNase treatment.

### Genome sequencing and annotation

Genomes and transcriptomes of both *P. macrospinosa* DSE2036 and *Cadophora* sp. DSE1049 were sequenced using the Illumina platform. For genomic sequencing, 500 ng of DNA was sheared to 270 bp using the covaris E210 (Covaris) and size selected using SPRI beads (Beckman Coulter). The fragments were treated with end-repair, A-tailing, and ligation of Illumina adapters using the TruSeq Sample Prep Kit (Illumina). For transcriptomics, stranded cDNA libraries were generated using the Illumina TruSeq Stranded RNA LT kit. mRNA was purified from 1 µg of total RNA using magnetic beads containing poly-T oligos, fragmented, and reversed transcribed using random hexamers and SSII (Invitrogen), followed by second strand synthesis. The fragmented cDNA was treated with end-pair, A-tailing, adapter ligation, and eight cycles of PCR.

All libraries were quantified using KAPA Biosystem’s next-generation sequencing library qPCR kit, run on a Roche LightCycler 480 real-time PCR instrument, and multiplexed into pools of two libraries. Samples were prepared for sequencing on the Illumina HiSeq sequencing platform using the TruSeq paired-end cluster kit v3, and Illumina’s cBot instrument to generate clustered flowcells. Sequencing of the flowcells was performed on the Illumina HiSeq. 2000 sequencer using a TruSeq SBS sequencing kit 200 cycles, v3, following a 2 × 150 indexed run recipe.

Genomic reads were initially assembled with two sets of assembly parameters using Velvet^52^. Simulated 2-kbp and 3-kbp-long mate-pair libraries were derived from these initial Velvet assemblies and combined with the original set of reads using AllPathsLG release version R47710^53^. Mitochondria were separately assembled with Velvet and then AllPathsLG to produce single mitochondrial contigs for each genome. Illumina reads of stranded RNA-seq data were used as the input for *de novo* assembly of RNA contigs. Reads were assembled into consensus sequences using Rnnotator (v. 3.3.2)^54^.

Both genomes were annotated using the JGI Annotation Pipeline^55^ and made available via the JGI fungal portal MycoCosm^55^. The genomes of *Cadophora* sp. and *P. macropsinosa* can be accessed at http://genome.jgi.doe.gov/Cadsp1 and http://genome.jgi.doe.gov/Perma1 respectively, and these Whole Genome Shotgun projects have been deposited at DDBJ/ENA/GenBank under the accession *Cadophora* sp., PCYN00000000 and *Periconia macrospinosa*, PCYO00000000.

### Clustering/phylogenomic analyses

For comparison of our two DSEs to other fungi, available genomes of 32 ascomycetes with different lifestyles including saprotrophic, mutualistic or pathogenic species were selected (Supplementary Table S1). Non-redundant predicted protein sequences from the genomes of 34 fungi species were clustered based on similarity using Mcl 14-137^56^ based on a similarity metric derived from blast score and alignment coverage as described in Ohm *et al*.^30^. An inflation parameter of 2.0 was used for clustering. To obtain sequence similarity measures, we use all-vs-all blastp as implemented in mpiBLAST 1.6.0, using an E-value cut-off of 10 (the default value).

To infer an organismal phylogeny, we identified single copy clusters that contained a single protein per species and included representatives of at least 25 species. Multiple alignments for these clusters were obtained using PRANK v.140603^57^. Ambiguously aligned regions of the alignments were removed with GBlocks 0.91b^58^ using the default, stringent settings. Next, we concatenated protein family alignments into a supermatrix, excluding any alignment having less than 50 amino acid residues. This resulted in a supermatrix of 929 protein families and 169,432 amino acid sites. Maximum Likelihood trees were inferred from this alignment using the WAG model of sequence evolution with gamma-distributed rate heterogeneity in RAxML 8.1.3^59^. and bootstrap support estimated in 100 replicates (Supplementary Fig. S1).

### COMPARE-analysis

We analyzed the genome evolution of DSEs and related ascomycetes by reconstructing gene duplication and loss histories across all recognized gene families in the 34 species listed in Supplementary Table S1. To this end, we aligned each protein cluster as above, and inferred ML gene trees in RAxML 8.1.3 under the WAG model with gamma-distributed rate heterogeneity. Gene trees were then reconciled with the species tree by TreeFix v1.1.10^60^ using 100 iterations and RAxML as the tree inference algorithm and the default duplication/loss cost model. We then identified protein orthogroups within reconciled gene trees using the ortholog coding algorithm^61^ and mapped the origins and losses of the resulting orthogroups on the organismal phylogeny using Dollo parsimony. For functional characterization of the duplication and loss events, we used Pfam domains and gene ontology terms. Pfam domains were scanned using PfamScan.pl, which utilized Hmmer version 3.1b1. We detected at least one known Pfam domain in 263,141 proteins (64.2%). For enrichment analyses, we used the hypergeometric test with Bonferroni correction for multiple hypothesis testing.

### CAZymes

We screened for carbohydrate-active enzymes (CAZymes) within the 34 genomes used in this study and also the three further genomes of DSE fungi, *Harpophora oryzae*^36^, *Phialocephala subalpina*^29^ and *Microdochium bolleyi*^37^ published recently. The detection, and family assignment of all CAZymes were performed as previously described^62–64^. BLAST and Hmmer searches were conducted against sequence libraries and Hmm profiles in the CAZy database (http://www.cazy.org). All positive hits were manually examined for final validation. We took into account all CAZyme classes, including Glycoside Hydrolases (GH), Carbohydrate Esterases (CE), Glycoside Transferases (GT), Polysaccharide Lyases (PL), Carbohydrate-Binding Modules (CBM) and Auxiliary redox enzymes (AA), both of which are thought to break down cell wall components, including lignin^62^. CAZymes selection for PCWDE were carried out using the CAZy database pipeline^62^. The copy numbers of each subfamilies of CAZyme families and PCWDEs were used as dataset for principal component analyses (PCA).

### Meiosis-related genes

Since teleomorphs and sexual state have never been observed in *P. macrospinosa* and *Cadophora* sp., we examined their genomes for meiosis-related genes. Protein sequences for the genes involved in meiosis were checked as described in Toome *et al*.^65^. Protein sequences were obtained from the Saccharomyces Genome Database^66^, and subjected to BLAST searches using the *Cadophora* sp. and *P. macrospinosa* genomes from MycoCosm^55^ as a reference. We searched for the presence of the core meiotic genes according to Halary *et al*.^67^ in all of the 34 genomes analyzed.

### Small secreted proteins

We screened for secreted proteins in *Cadophora* sp. and *P. macrospinosa* using the SignalP 4.1 server^68^ with default settings for eukaryotic organisms. Putative small secreted proteins (SSP) were predicted considering peptides both with and without trans-membrane segments. Signal peptides between 80–300 amino acids were considered as SSPs.

### Genes encoding melanin synthesis pathway proteins

Based on literature data^69,70^, we searched for proteins involved in melanin biosynthesis using BLAST. We focused on DHN-melanin synthesis – the main form of melanin produced by ascomycetes – and categorized protein sequences according to Tsai *et al*.:^69^ *Alb1 *– polyketide synthase AAC39471.1; *Arp1 *– scytalone dehydratase (PF02982) AAC49843.1; *Arp2 *– 1,3,6,8-tetrahydroxynaphthalene (THN) reductase AAF03314.1; *Abr1* – brown 1 AAF03353.1; *Ayg1 *– yellowish-green 1 AAF03354.1; *Abr2* – brown 2 AAF03349.1. *Abr1* and *Abr2* were not separated, but discussed together as *Abr1–2* due to highly overlapping results.

### Aquaporins

We searched for annotations of aquaporins in the MycoCosm resource to collect the sequences of major intrinsic proteins (MIP) – both aquaporin and aquaporin-like genes. Non-MIPs (Cadsp_426096) and proteins without Pfam domains (Perma_660761 and Perma_725695) were excluded from further analyses. For phylogenetic analysis of aquaporins, we merged the collected protein sequences with the fungal aquaporin dataset of Xu *et al*.^71^. Following the classification of Verma *et al*.^72^, we categorized the proteins into main groups of aquaporins: AQP, AQGP and its subgroup XIP. Maximum Likelihood analysis of aquaporins was carried out with raxmlGUI v. 1.3^73^ running RAxML 8.1.3^59^ and ML bootstrap analysis with 1,000 replicates. The phylogenetic tree was visualized and edited using MEGA6^74^.

### Secreted peptidases and lipases

To assess the diversity of secreted peptidases and lipases, we collected genes using the search terms “protease or peptidase” and “lipase” in MycoCosm. Prediction of secretion signals was conducted using SignalP^75^ and analyzed on the SignalP 4.1 server using the default settings for eukaryotic organisms. Secreted protease sequences were used in BLASTp searches (e-value cut-off = 1e-04) against the MEROPS database^76^ (http://merops.sanger.ac.uk/): aspartic (A), cysteine (C), serine (S), metallo (M) and threonine (T) peptidases, class with unknown activities (U) and peptidase inhibitors (I) were separated. Putative secreted lipases of the 34 fungi were classified according to their best hits in a BLASTp (e-value cut-off = 1e-04) search against the Lipase Engineering Database (http://www.led.uni-stuttgart.de/). Secreted lipase protein sequences were grouped into the main classes and their superfamilies. Protein sequences of both types of enzyme were cross checked with a BLAST search (e-value cut-off = 1e-04) against the NCBI NR protein database.

## Results and Discussion

### Genomes

**Figure 2:**
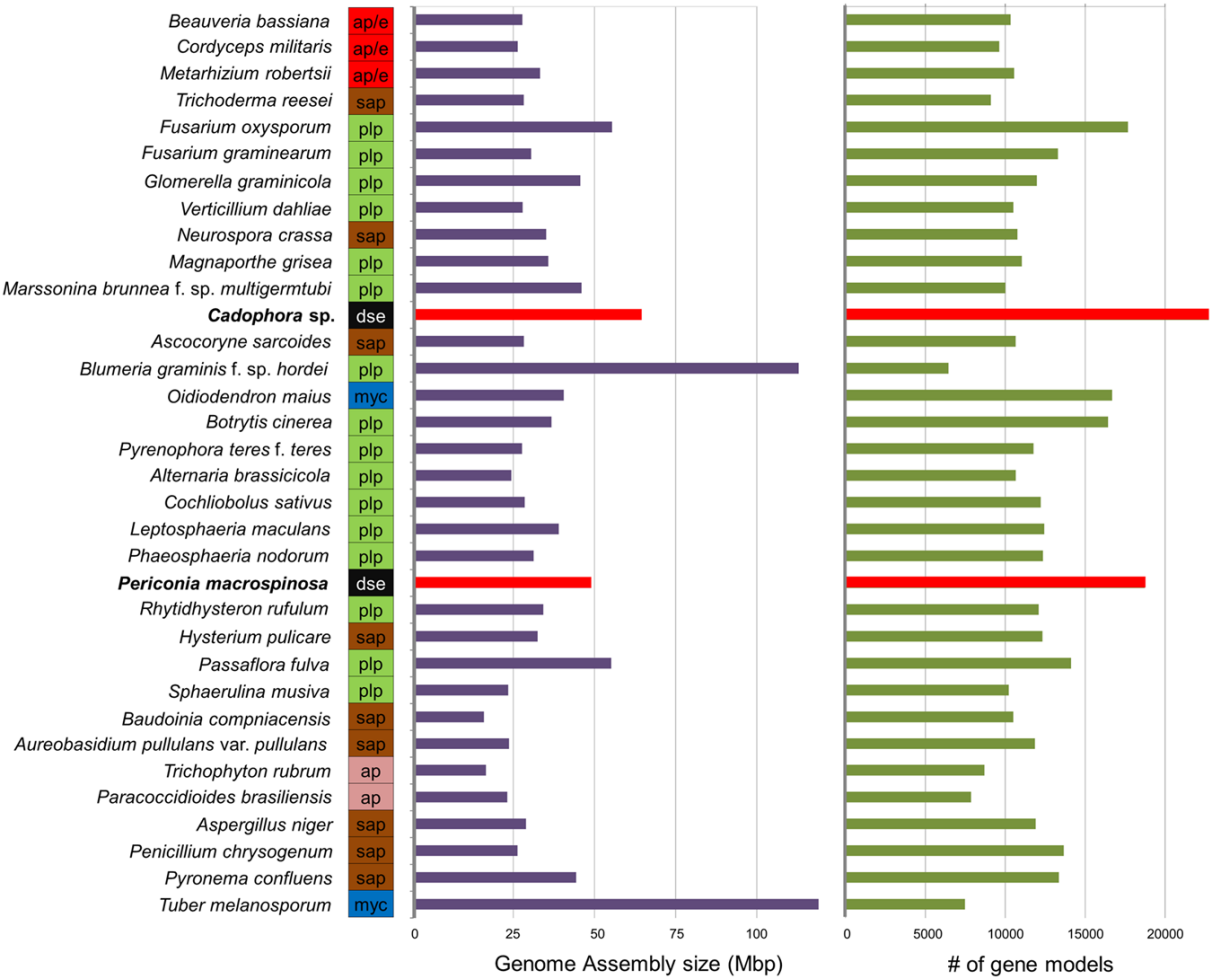
Genome size and number of predicted genes of *Cadophora* sp. and *Periconia macrospinosa* as compared with other ascomycetes. DSEs are labeled with bold names and red bars. After each species name, its type of lifestyle is indicated as follows: red (ap/e; animal pathogens/endophytes?), brown (sap; saprotrophs), green (plp; plant pathogens), black (dse, dark septate endophytes), blue (myc; mycorrhizal fungi), or pink (ap; animal pathogen). For complete species names and further information see Supplementary Table S1.

In the present study, we sequenced the genomes of two common DSEs, *Cadophora* sp. and *Periconia macrospinosa*, originating from the same semiarid grassland habitat (Table 1). *Cadophora* sp. has relatively big genome size of 70.46 Mb (GC content: 45.82%) including 22,766 gene models, and *P. macrospinosa* has still large, 54.99 Mb genome (GC content: 47.24%) with 18,750 models (Fig. 2; Table 1). In comparison with other ascomycetes included in this study, the two DSEs, especially *Cadophora* sp., have larger genome sizes and higher numbers of predicted proteins (Fig. 2; Supplementary Table S1). The number of gene models of functionally classified proteins (KOG) is similar between the *Cadophora* sp. and *P. macrospinosa* genomes (Supplementary Fig. S2). The genomes of both DSEs are also larger than the average genome size of previously sequenced ascomycetous species^77^, and *Cadophora* sp. is similar in size to *Phialocephala subalpina* (79.7 Mb), a common DSE in forest ecosystems^29^. The larger genome size of *Cadophora* sp. and *P. macrospinosa* is caused by expansion of the protein coding gene inventory, and not by transposable element (TE) proliferation, as seen in several fungi^78,79^, causing genome size expansion in several fungi and oomycetes such as the pathogens *Phytophthora infestans*^80^, *Blumeria graminis* f.sp. *hordei*^81^ and *Leptosphaeria* species^82^, and the ectomycorrhizal ascomycete *Cenococcum geophilum*^83^.

**Table 1:** Genome statistics of *Cadophora* sp. DSE1049 and *Periconia macrospinosa* DSE2036.

Genome Assembly		Cadophora sp.	Periconia macrospinosa
	Genome Assembly size (Mbp)	70,46	54,99
	Sequencing read coverage depth	79,2x	139,4x
	# of contigs	1294	2470
	# of scaffolds	1193	1566
	# of scaffolds > = 2Kbp	1092	1217
	Scaffold N50	71	101
	Scaffold L50 (Mbp)	0,24	0,14
	# of gaps	101	904
	% of scaffold length in gaps	0,001	0,018
	Three largest Scaffolds (Mbp)	1,38; 1,24; 1,23	1.32, 0.77, 0.70
External gene models/length (bp)			
Average	gene	1583	1564
	transcript	1401	1418
	exon	460	537
	intron	91	91
Median	gene	1358	1340
	transcript	1200	1202
	exon	292	338
	intron	58	61
Description			
Average	protein length (aa)	409	411
	exons per gene	3,04	2,64
	# of gene models	22766	18750
Median	protein length (aa)	338	337
	exons per gene	3	2
CEGMA	%	100	100
ESTs			
	# sequences total	39120	40915
	# mapped to genome	38428	38019
	% mapped to genome	0,982	0,929

### Reconstructing genome evolution in DSEs

**Figure 3:**
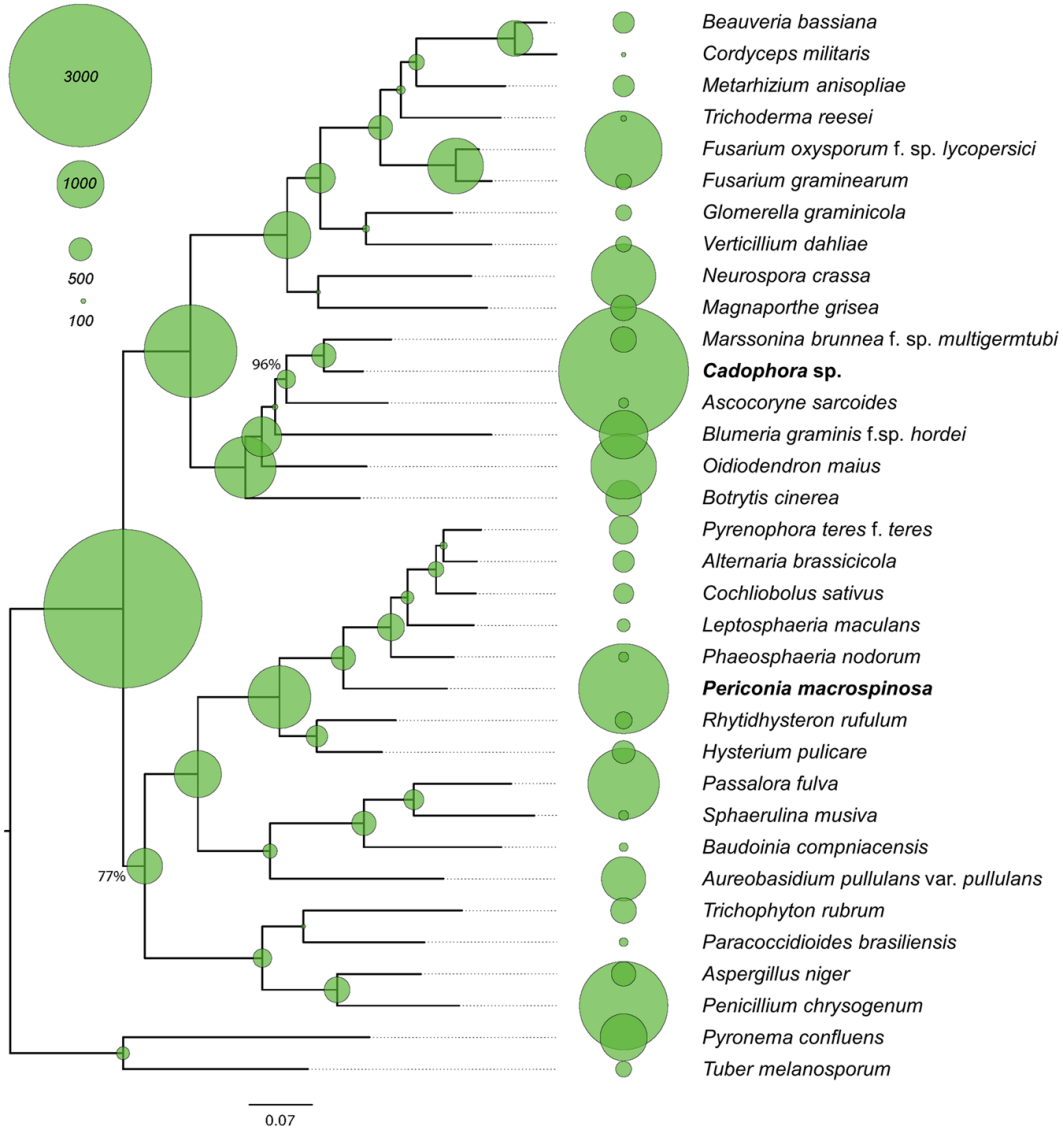
Genome-wide reconstruction of gene duplication histories of *Cadophora* sp., *Periconia macrospinosa* and another 32 ascomycetes. Green circles indicate observed (on terminal branches) and reconstructed (on internal nodes) copy numbers. The two DSEs are marked in bold.

Markov clustering resulted in 21,768 clusters (at I = 2.0), from which patterns of genome evolution were inferred (Fig. 3). We found 9,478 clusters that did not contain any DSE fungi. These included large clusters of permeases, transferases and other enzymes related to common cellular processes of plant associated fungi (Supplementary Table S2). A total of 272 and 211 clusters were specific for *Cadophora* sp. and *P. macrospinosa*, respectively, while only 26 clusters contained proteins of the two DSEs only. Interestingly, for both species there is a high number of protein clusters that do not contain any known Pfam domains (>55%). The lack of functional annotation for the majority of DSE-specific clusters shows that many of the proteins of these fungi have no similarity to the characterized fraction of the protein space. This could imply DSE fungi have evolved unique genetic innovations.

Only twelve of the 26 clusters shared by both DSEs contained known Pfams (Supplementary Table S2). The largest DSE-specific clusters contained genes of heterokaryon incompatibility (HET), toxicity, transferase and kinase activities and unknown function (Supplementary Table S3). Surprisingly, HET genes were highly expanded in the DSE genomes, especially in *Cadophora*. We detected significantly more HET genes (using the search term PF06985) in *Cadophora* sp. (470) and *P. macrospinosa* (177) than in other Ascomycota (57 genes on average, Supplementary Table S3). Genes containing the functionally uncharacterized domain PF12013 are also expanded in the DSE genomes. This domain, which is also found in other ascomycetes and eukaryotes, contains two segments that are likely to be C_2_H_2_ zinc-binding domains. Furthermore, the NACHT domain (PF05729), which is linked to HET proteins and is likely responsible for vegetative incompatibility, is also overrepresented in DSE genomes (p < 0.001, Fisher’s exact test, Supplementary Table S3). Although our knowledge of the HET genes and their biological significance in filamentous fungi is still limited^84^, their role in (non-)self-recognition and hyphal fusion could imply that DSEs need particularly sophisticated mechanisms for managing intra- or interspecies hyphal encounters^85^.

Of the species-specific clusters of *Cadophora* sp. (272) and *P. macrospinosa* (211) 35 and 26 contained known Pfam domains, respectively (Supplementary Table S2). These protein clusters exhibit different functions, including transferase and kinase activities. While the largest cluster specific to *Cadophora* sp. with a Pfam comprised 46 endonucleases of the DDE superfamily (PF13358), the largest cluster specific to *P. macrospinosa* with a Pfam comprised HET domain-containing proteins (Supplementary Table S2).

We analyzed gene duplication/loss events in two DSEs and 32 other ascomycetes with diverse lifestyles. Altogether, 13,966 reconciled gene trees, supplemented with 6,991 clusters that contained less than four proteins were used for mapping gene duplications and losses. We found that 65,443 gene duplications and 194,331 gene losses were required to explain genome evolution in the examined species under Dollo parsimony. The inferred numbers of duplications and losses, as well as the reconstructed ancestral genome sizes, are shown in Fig. 3. Both DSEs showed high numbers of species-specific gene duplications: 2,757 and 1,931 in *Cadophora* sp. and *P. macrospinosa*, respectively (Fig. 3). Enrichment analyses on the gene families that showed duplications in *Cadophora* sp., in *P. macrospinosa*, or both species revealed that several terms related to oxidation-reduction processes, peptidase and kinase activities, and transmembrane transport were enriched (Supplementary Table S3). On the *Cadophora* sp. branch, we inferred a total of 2,757 gene duplications in 1,329 clusters. Of these, 221 clusters were species-specific, but only 14 of them contained Pfams, which had six GO terms overrepresented. In *P. macrospinosa*, we inferred 1,931 duplications in 940 clusters. *P. macrospinosa* had 155 species-specific clusters, of which 14 also had Pfams. We found 131 clusters showing duplications in both species, of which 84 had Pfams (Supplementary Table S2). Enrichment analysis also revealed clusters in which *Cadophora* sp., *P. macrospinosa*, or both fungi lost genes. While *Cadophora* sp. lost gene clusters that comprised a total of ten GO terms, *P. macrospinosa* lost even more clusters that added up to 63 GO terms. The two DSEs lost nine shared gene clusters (Supplementary Table S2).

Taken together, analyses of the DSE genomes show significant expansions of certain gene families, including a high number of species-specific gene duplications in both *Cadophora* sp. and *P. macrospinosa*. This, together with low levels of convergence in gene family evolution, suggests that, despite originating from the same habitat, these two DSEs evolved along different evolutionary trajectories and display considerable functional differences.

### CAZymes

**Figure 4:**
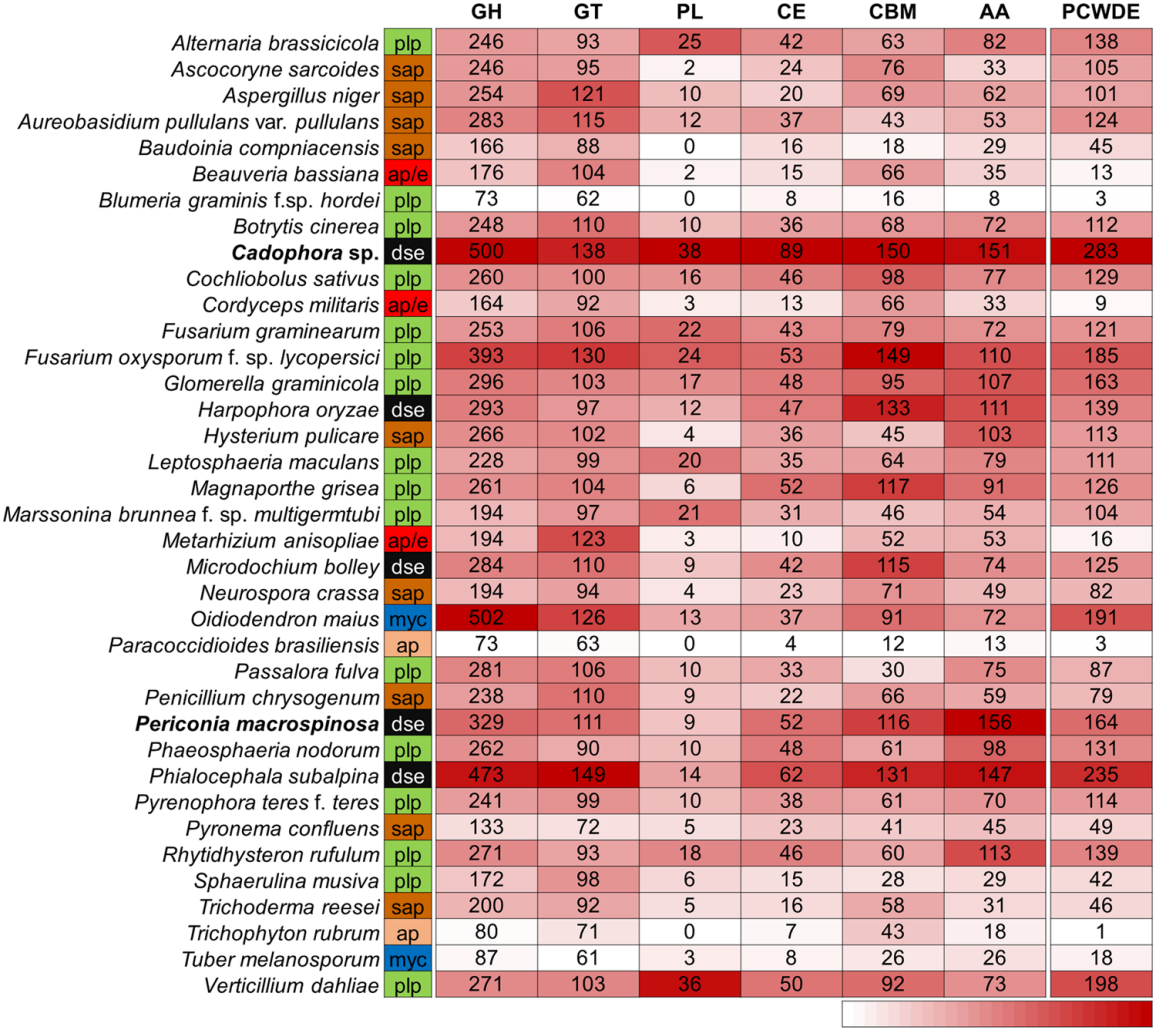
Number of genes encoding carbohydrate active enzymes (CAZymes) and plant cell wall degrading enzymes (PCWDE) in *Cadophora* sp., *Periconia macrospinosa* and other 35 fungi including three further DSE species. Major CAZyme classes are shown separately, including Glycoside Hydrolases (GH), Glycoside Transferases (GT), Polysaccharide Lyases (PL), Carbohydrate Esterases (CE), Carbohydrate-Binding Modules (CBM), and Auxiliary redox enzymes (AA). After each species name, its type of lifestyle is indicated as follows: red (ap/e; animal pathogens/endophytes), brown (sap; saprotrophs), green (plp; plant pathogens), black (dse, dark septate endophytes), blue (myc; mycorrhizal fungi), or pink (ap; animal pathogen). For each class of enzyme, white-to-red shading corresponds to lower to higher copy numbers. For detailed information on CAZymes copy numbers see Supplementary Table S4.

**Figure 5:**
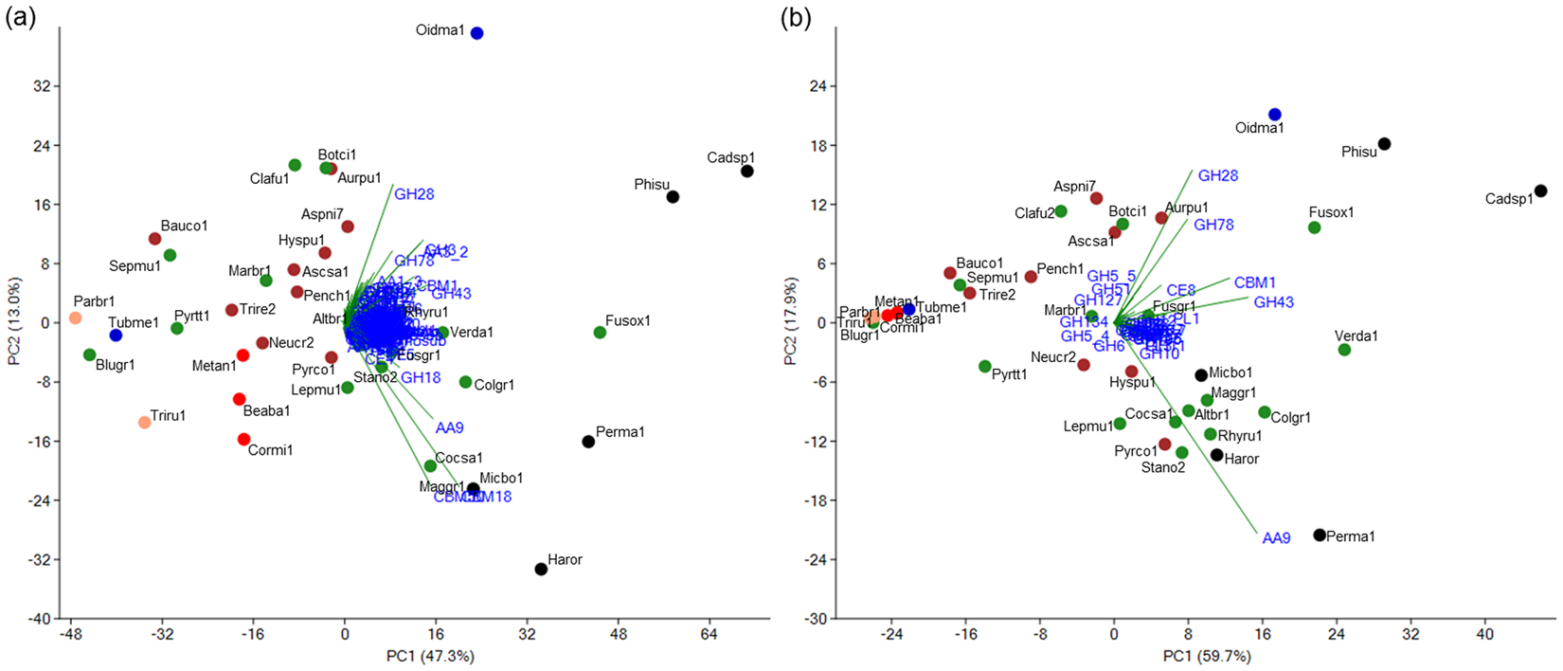
Principal component analysis (PCA) of carbohydrate active enzymes (CAZymes) and plant cell wall degrading enzymes (PCWDEs) of *Cadophora* sp., *Periconia macrospinosa*, and 35 other ascomycetes including three further DSE species. (**a**) PCA based on CAZyme copynumbers. PC1 accounts for 47.3% of the variation and PC2 for 13%. (**b**) PCA based on gene copy numbers of plant cell wall degrading families. PC1 accounts for 59.7% of the variation and PC2 for 17.9%. The different fungal lifestyles are labelled in red (ap/e; animal pathogens/endophytes?), brown (sap; saprotrophs), green (plp; plant pathogens), black (dse, dark septate endophytes), blue (myc; mycorrhizal fungi), or pink (ap; animal pathogen). For complete species names and further information see Supplementary Table S5.

The genomes of *Cadophora* sp. and *P. macrospinosa* contained 1,066 and 773 genes encoding putative CAZymes, respectively (Fig. 4; Supplementary Table S4). These numbers are significantly higher than the average for the other 32 species in our analysis, even when the effect of genome-size differences is taken into account (p < 0.001, Fisher’s exact test, Supplementary Table S3). We found *Cadophora* sp. to have the highest number of CAZymes, followed by *Phialocephala subalpina* (976), *Fusarium oxysporum* (859), *Oidiodendron maius* (841), *P. macrospinosa*, *Harpophora oryzae* (693), *Glomerella graminicola* (666) and *Microdochium bolleyi* (634) (Fig. 4; Supplementary Table S4). All five DSE fungi analyzed were among the first eight taxa with highest number of CAZymes (Fig. 4; Supplementary Table S4) Principal component analysis (PCA) based on CAZymes separated the five DSEs and the root colonizing *F. oxysporum*, which also has endophytic stage in its life cycle from all the other taxa (Fig. 5a). The GH superfamily is the most represented class of CAZymes found within our two DSE genomes, with the enlarged families being GH3 (b-glucosidase/b-xylosidase), GH16 (b-galactosidase/b-glucanase), GH18 (chitinases) and GH43 (a-arabinofuranosidase/b-xylosidase). We also found high gene numbers of GH78 (a-rhamnosidase) family, but only in *Cadophora* sp. In general, the DSEs possessed relatively high number of genes in the CAZyme superfamilies and families/subfamilies across the Ascomycota taxa examined. This was the case, for example, for CBM1, CBM18 and CBM50 among the CBMs, and AA3_2 and AA9 among the AAs (Supplementary Table S4).

We also found *Cadophora* sp. and *P. macrospinosa* to be enriched (p value < 0.001, Fisher’s exact test, Supplementary Table S3) in proteins with PCWDE domains (283 and 164, respectively), and a PCA based on PCWDEs separated our DSEs from other species (Fig. 5b). *Cadophora* sp. has the highest number of PCWDE genes out of all ascomycetes analyzed in this study (Supplementary Table S5). This species has even more PCWDEs than the pathogenic *Colletotrichum* species (>240)^83,86,87^, and the other helotialean DSE, *P. subalpina* (235) (Supplementary Table S5). Even though the most abundant PCWDE domains were the same (GH43, CBM1, and AA9) for both DSEs, the two species were separated from each other in the PCA (Fig. 5b). In this analysis the five DSE did not form a separate group as when the whole CAZomes were analyzed.

The fact that these DSEs possess a high number of CAZymes and PCWDE domains suggests that they have a particular and broad spectrum of degrading enzymes and possible plant cell wall degrading capacity. Several genomic studies showed that an expanded CAZyme and PCWDE repertoire was linked with saprobic and/or plant pathogenic abilities of fungi^30,38,88–90^. This is consistent with the fact that those fungi are able to break down complex plant polysaccharides - a feature that is important for establishing infection and accessing nutrients during necrotrophic and saprotrophic growth. The PCA of PCWDEs revealed a loose association between DSE fungi, root colonizing plant pathogens (*Fusarium oxysporum* and *Verticillium dahliae*) and the ericoid mycorrhizal fungus, *O. maius*, along the first axis (Fig. 5b). However, these species dispersed along the second axis, indicating that they have substantial differences with respect to PCWDE domains. Schlegel *et al*.^29^ found a similar expansion of CAZyme and PCWDE genes in the genome of *P. subalpina*. The copy numbers of PCWDEs in this fungus are quite similar to those in *Cadophora* sp. and the PCA also showed their similarity in PCWDEs, which might be consistent with their close phylogenetic relationship (Helotiales) and similar lifestyle (DSE). In our study and in the study of Schlegel *et al*.^29^, ectomycorrhizal species, which are generally characterized by a reduced CAZyme repertoire (especially of PCWDEs)^38^, showed fundamental differences from root endophytes in terms of these genes. The ectomycorrhizal lifestyle, which has arisen from saprobic ancestors multiple times, is linked to convergent loss of genes encoding PCWDEs^38^. Similarly, the non-root colonizing endophyte *X. heveae* was also found to have reduced spectra of these enzyme families^31^.

The number of CAZymes in the helotialean DSE is even higher than that in ericoid mycorrhizal species where saprobic capacities of the fungal partner has great importance^91^. The observation that all sequenced DSE fungi show signs of CAZyme family expansion suggests that PCW degradation may be one of the most important attributes of DSE fungi. These enzymes are undoubtedly involved in saprotrophic activity, and might be prerequisite for root colonization and interaction with the host plant.

### Meiosis-related genes

DSEs are generally considered as asexual fungi, and up until now, the *in vitro* induction of the sexual form (ascomata) has only ever been reported once for one genus^42^. Numerous homologs of meiosis-related genes could be identified in both the *Cadophora* sp. and *P. macrospinosa* genome: 93 and 89 of the 127 meiosis-related genes searched were found, respectively (Supplementary Table S6). However, out of the two genes (*ndt80* and *ime1*) considered to play a vital role in meiosis in *Saccharomyces cerevisiae*^92^, one or both were absent in *Cadophora* sp., and *P. macrospinosa*, respectively. Other genes considered as essential for meiosis in *S. cerevisiae* and other eukaryotes (e.g., *sum1* and *xrs2*) were also missing from the genomes of the two DSEs. Out of 31 meiosis genes which were previously determined as core genes^67^, 28 and 26 orthologues were found in *Cadophora* sp. (lacks *dmc1, hop1*, and *rad51*) and *P. macrospinosa* (lacks *dmc1, hop1, rad51, mus81* and *rec8*) genomes, respectively (Supplementary Table S6). Compared to the other 32 genomes representing both sexual and asexual ascomycetes, the lack of three and five core genes in DSE genomes is not exceptional even in fungi capable for sexual reproduction (Supplementary Table S6). So, the 73% and 70% of all meiosis-related genes (90% and 84% of all core meiosis genes *sensu* Halary *et al*.^67^) found in the *Cadophora* sp. and *P. macrospinosa* genomes are not unambiguous indicators of the the absence of potential meiotic processes. This might indicate previous existence of sexual reproduction in these DSEs and we cannot rule out the existence of cryptic sexual reproduction either. Probably, the expansion of the HET genes in these species is associated with the low selective pressure at the MAT locus, and just as proposed in case of black yeasts^93^, a parasexual cycle may play an important role in generating diversity.

### SSPs

Small secreted proteins are important players in fungal-plant interactions^41,78,94^. We predicted a total of 1,912 and 1,543 SSP genes in *Cadophora* sp. and *P. macrospinosa*, respectively, which represent significant percentages (8.4% and 8.2%) of the proteomes. Large number of the SSPs with no homology with previously identified SSPs is not surprising considering that the majority of known SSPs are taxon specific^38–41,83^. Multiple SSPs play a role in the symbiotic interactions of mycorrhizae^38,41^ and the root endophyte *Piriformospora indica*^34^, so we may assume that some of the predicted SSPs of the DSEs are symbiosis-related. Although the precise mechanisms by which *Cadophora* sp. and *P. macrospinosa* suppress plant defense are unknown, the SSPs predicted from their genomes could be candidate effectors^95^.

### Genes belonging to melanin synthesis pathways

Many fungal species produce pigments such as melanin, which is generally produced via the DHN-melanin synthesys pathway and plays crucial roles in an array of cellular processes, including defense or pathogenicity^96^. We searched for homologs of the genes of the DHN-melanin synthesis pathway, namely *Alb1*, *Arp1*, *Arp2*, *Abr1–2* and *Ayg1*, in all 34 fungi. As expected, high numbers of melanin synthesis related genes were found in the pigmented ascomycetes, including the ericoid mycorrhizal *O. maius*, but also in the two DSEs (Supplementary Table S7). The total numbers of genes related to DHN-melanin synthesis were similar in all three of these species: 134 genes in *Cadophora* sp., 133 in *P. macrospinosa*, and 151 in *O. maius*. However, *Cadophora* sp. and *P. macrospinosa* differed with respect to the most dominant melanin synthesys-related genes. For example, although *Alb1* homologues (PKSP) were overrepresented in both DSEs (p < 0.001, Fisher’s exact test, Supplementary Table S3), their number was higher in *P. macrospinosa* (67 genes). In contrast, *Cadophora* sp. possessed more *Arp1* (scytalone dehydratase)*, Arp2* (THN reductase) and *Abr1–2* homologues (3, 79 and 27, respectively) (Supplementary Table S7). It has been demonstrated that these pigments serve to protect fungal cells, especially from reactive oxygen species produced by host immune defenses^97^. Moreover, as pigments can be advantageous to plant-associated fungi in habitats with strong UV-radiation, which is consistent with the high numbers of homologues identified in grassland-inhabiting DSE fungi.

### Aquaporins

We found 15 and 9 MIP genes in the *Cadophora* sp. and *P. macrospinosa* genomes, respectively. Out of all the genomes screened in this study, *Cadophora* sp. had the most MIP genes (Supplementary Table S8). Although the majority of the MIPs were aquaporins, aquaglyceroporins and X-intrinsic proteins were also present. The aquaporin gene tree shows that the majority of aquaporin genes found in the two DSEs belong to known groups of aquaporins. Nonetheless, two new lineages of aquaporins were identified from DSE genomes (Supplementary Fig. S3). The expansion of aquaporin genes, along with potentially novel clades in *Cadophora* sp., could suggest a major role for these proteins in DSE fungi. Whether these genes are upregulated during the symbiotic phase in DSE fungi, as reported in EcM fungi^40,83^, represents an interesting future research question. Gene numbers may indicate some similarities with ectomycorrhizal fungi in the aquaporin-related MIPs^40^.

### Secreted peptidases and lipases

The total number of genes encoding secreted peptidases in *Cadophora* sp. was enlarged, however only the aspartic peptidase family were significantly higher than average (p < 0.001, Fisher’s exact test, Supplementary Table S3) when compared to other ascomycetes (Supplementary Table S9). In general, plant-associated fungi had more secreted peptidase genes than fungi with other lifestyle in our analysis. Accordingly, PCA grouped DSE fungi with other plant-associated species including *O. maius*, plant pathogens, and the three hypocrealean entomopathogen/endophyte species (Supplementary Fig. S4a). *Cadophora* sp. was enriched in secreted lipases (48) (p < 0.001, Fisher’s exact test, Supplementary Table S3) compared to the 33 species analyzed in this study (Supplementary Fig. S4b; Supplementary Table S9), followed by *O. maius* (30) and *P. macrospinosa* (29). Separation of DSE fungi from the other species along the PC1 axis was correlated with abH03 superfamily copy numbers (*Candida rugosa* lipase like) (Supplementary Fig. S4b).

Since all the plant-associated fungi in this study, except *T. melanosporum*, had genomes enriched with secreted peptidases and lipases, these enzymes are likely to be important for plant colonization by DSEs. Compared to saprobes and human pathogens, peptidases are more abundant in these fungi mainly due to expansions in the serine protease and metalloprotease families. A similar trend has previously been observed in the plant pathogenic *Colletotrichum* spp^86,98^. The majority of secreted proteases in *Cadophora* sp. were subtilisins (S8A), a family of serine proteases associated with virulence, penetration and colonization of hosts^99,100^. On the other hand, the two DSE fungi exhibit different patterns of metallopeptidases copy numbers. For instance, while M43 proteases were the most abundant metallopeptidase family in *Cadophora* sp, they were completely absent in *P. macrospinosa*. These metallopeptidases are also expanded in *Piriformospora indica*, and their upregulation during colonization of dead roots^34^, which supports a role for proteases in degrading plant tissues.

Secreted lipases were overrepresented in the two DSEs, in other plant-associated fungi, and in entomopathogenic/endophytic species, compared to other Ascomycota (Supplementary Table S10). This is not surprising considering that several lipases are known to play important roles in plant pathogenicity. By catalyzing the hydrolysis of ester bonds of the fatty acid polymers in the plant cuticle, these lipases facilitate fungal penetration^101^. Secreted lipases are likely to serve as virulence factors in the colonization of arthropods^102^ and in fungal-plant interactions^101^. Moreover, lipases are highly expressed symbiosis-related factors involved in ectomycorrhizal symbiosis^40^. For example, the third most expressed gene in *T. melanosporum* during EcM symbiosis^40^ was a secreted lipase. Importantly, several homologs of this same lipase were in both DSEs, indicating its possible role in root colonization by endophytes.

## Conclusions

In this study, we analyzed the genomes of two independently evolved DSE fungi, which originated from the same environment. In comparison to other ascomycetes, we found that the DSEs have an increased genome size, a larger gene repertoire, and an expanded number of CAZymes, including PCWDEs. Aside from these common fundamental features, we also found major differences between the two fungi. Based on gene copy numbers, *Cadophora* sp. has a larger toolbox for saprobic capacity. According to DSE-specific gene clusters – both shared and unique ones – endophytes have no common DSE-specific function, but rather diverse roles. We found that all sequenced DSE fungi show signs of CAZyme family expansion. The complex carbohydrate degrading capacity could be a key characteristic of the lifestyle of DSE fungi.

Genome-wide reconstruction of gene duplication and loss histories revealed high numbers of species-specific gene duplications in the two DSEs and low levels of convergence in gene family evolution. This too confirms the striking functional diversity among DSE fungi. The results of our comparative genomics analyses reinforce this apparent diversity, and imply that an endophytic lifestyle does not comprise a homogenous ecological guild. As previously hypothesized^23^, functional diversity could be the key aspect of DSE function, and this complementarity might play a role in completing the plant holobiont^103^ and ensuring survival in nutrient-limited environments.

## Electronic supplementary material

Supplementary Figures

Dataset 1

Dataset 2

Dataset 3

Dataset 4

Dataset 5

Dataset 6

Dataset 7

Dataset 8

Dataset 9

Dataset 10
