# Supplementary material for: Comparative genomics provides insights into the lifestyle and reveals functional heterogeneity of dark septate endophytic fungi
Source: Sci Rep. 2018 Apr 20;8:6321. doi: 10.1038/s41598-018-24686-4 (PMC5910433; doi:10.1038/s41598-018-24686-4)
Supplement: Supplementary file 1 — Supplementary Figures [file 41598_2018_24686_MOESM1_ESM.pdf]

Supplementary Information

Comparative genomics provides insights into the lifestyle and reveals functional heterogeneity of dark septate endophytic fungi

Dániel G. Knapp[1], Julianna B. Németh[1], Kerrie Barry[2], Matthieu Hainaut[3,4], Bernard Henrissat[3,4,5], Jenifer Johnson[2], Alan Kuo[2], Joanne Hui Ping Lim[2], Anna Lipzen[2], Matt Nolan[2], Robin A. Ohm[2,6], László Tamás[7], Igor V. Grigoriev[2,8], Joseph W. Spatafora[9], László G. Nagy[10], Gábor M. Kovács[1,11],*

[1]Department of Plant Anatomy, Institute of Biology, Eötvös Loránd University, Budapest 1117, Hungary; [2]US Department of Energy (DOE) Joint Genome Institute, Walnut Creek CA 94598, United States; [3]Architecture et Fonction des Macromolécules Biologiques (AFMB), UMR 7257 CNRS Université Aix-Marseille, 13288 Marseille, France; [4]INRA, USC 1408 AFMB, 13288, Marseille, France; [5]Department of Biological Sciences, King Abdulaziz University, Jeddah, 21589, Saudi Arabia; [6]Microbiology, Department of Biology, Faculty of Science, Utrecht University, Utrecht, The Netherlands; [7]Department of Plant Physiology and Molecular Plant Biology, Institute of Biology, Eötvös Loránd University, Budapest 1117, Hungary; [8]Department of Plant and Microbial Biology, University of California Berkeley, Berkeley, CA, 94720, USA; [9]Department of Botany and Plant Pathology, Oregon State University, Corvallis, United States; [10]Synthetic and Systems Biology Unit, Institute of Biochemistry, BRC-HAS, Szeged 6726, Hungary; [11]Plant Protection Institute, Centre for Agricultural Research, Hungarian Academy of Sciences, Budapest 1022, Hungary.

* Correspondence and requests for materials should be addressed to G.M.K (gmkovacs@caesar.elte.hu)

SUPPLEMENTARY FIGURES

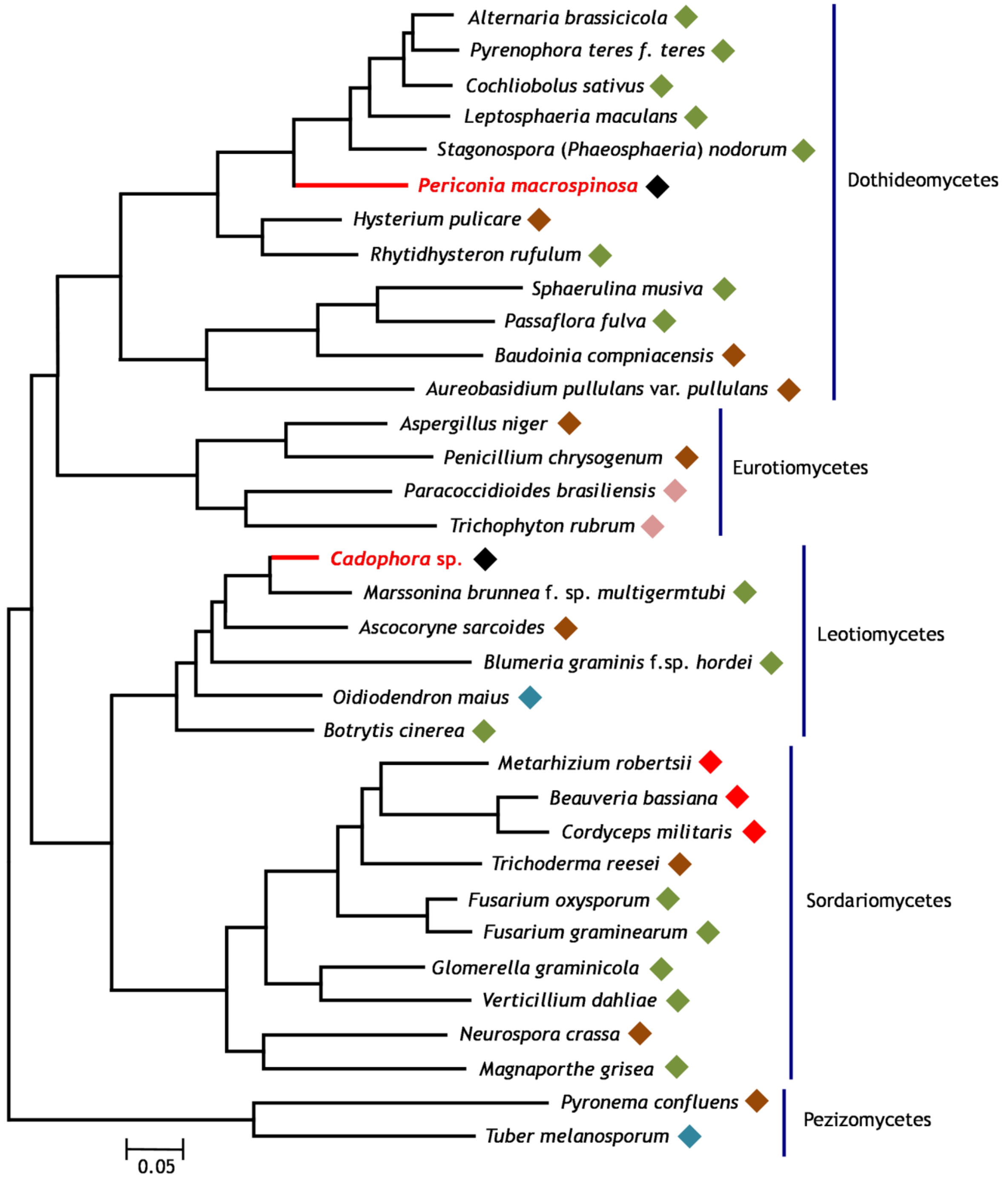

Supplementary Figure S1. Organismal tree of 34 species comprised in the study. The phylogram inferred from concatenated sequence alignment of singlecopy clusters (929 protein families) with 169432 amino acid sites. ML trees were inferred using the WAG model with gamma-distributed rate heterogeneity in RAxML 8.1.3. Colors indicate different lifestyles according to Supplementary Table S1.

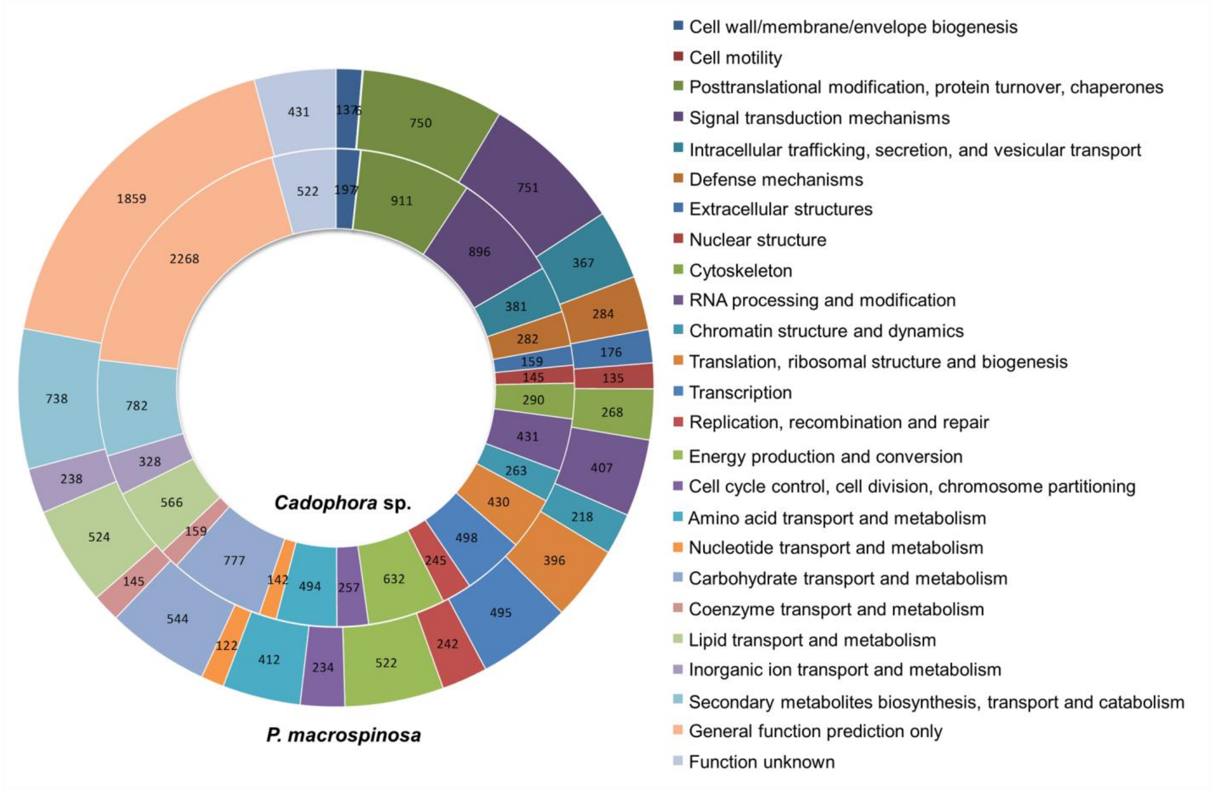

Supplementary Figure S2. Functional classification and comparison of *Cadophora* sp. and *Periconia macrospinosa* proteins. Each segment represents the relative fraction of genes pertaining to a specific functional category for the corresponding genome. The numbers of genes are also shown in each segment.

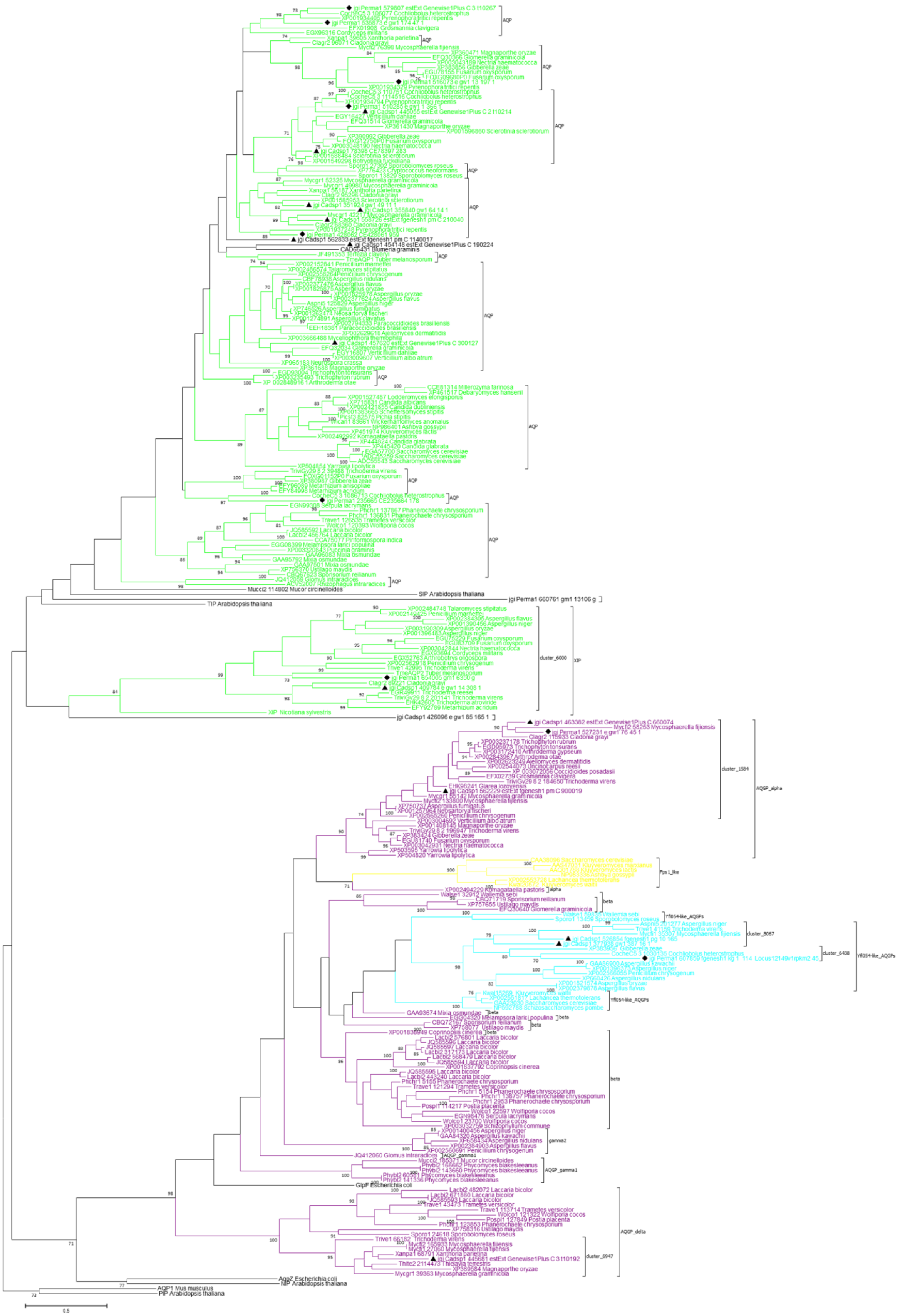

AQP
cluster_6000
XIP
cluster_1584
AQGP_alpha
Fps1_like
alpha
beta
Yfl054-like_AQGPs
cluster_8067
cluster_6438
gamma2
AQGP_gamma1
AQGP_delta
cluster_6947
SIP Arabidopsis thaliana
TIP Arabidopsis thaliana
GlpF Escherichia coli
AqpZ Escherichia coli
AQP1 Mus musculus
NIP Arabidopsis thaliana
PIP Arabidopsis thaliana
0.5

Supplementary Figure S3. Maximum Likelihood (RAxML) tree of representative sequences of aquaporin (AQP) families from the dataset of Xu *et al*.[71] supplemented with the AQP sequences of *Cadophora* sp. and *Periconia macrospinosa*. RAxML bootstrap support values obtained from 500 replicates are shown (≥ 70) at the branches. Different AQP families are shown in different colors, and sequences of the two DSEs are labeled with symbols. The scale indicates five expected changes in 100 characters.

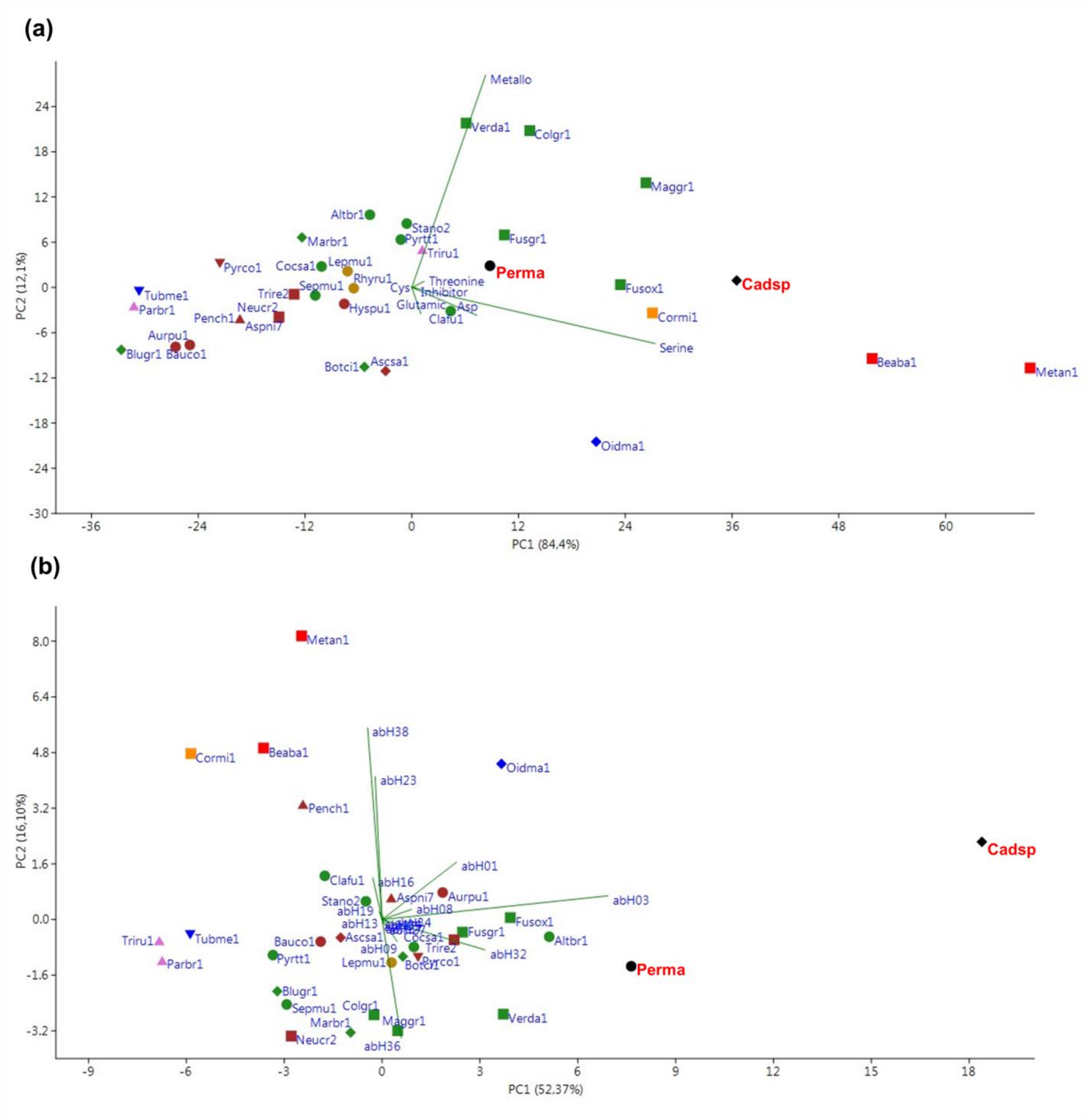

Supplementary Figure S4. Principal component analysis (PCA) based on secreted proteases and lipases of *Cadophora* sp., *Periconia macrospinosa* and other ascomycetes. (a) PCA of the number of genes of different secreted protease families of the 34 ascomycetes. PC1 accounts for 84.4% of the variation and PC2 for 12.1 %. (b) PCA of gene copy numbers of different gene families of secreted lipases. PC1 accounts for 52.4 % of the variation and PC2 for 16.1%. The different fungal lifestyles are labelled in red (ap/e; animal pathogens/endophytes?), brown (sap; saprotrophs), green (plp; plant pathogens), black (dse, dark septate endophytes), blue (myc; mycorrhizal fungi), or pink (animal pathogen). Matching symbols are used to indicate fungi belonging to the same ascomycetous order. Matching symbols are used to indicate fungi belonging to the same ascomycetous order. Abbreviations for taxon names are defined in Supplementary Table S1.
